# Buzz Kill: Function and Proteomic Composition of Venom from the Giant Assassin Fly *Dolopus genitalis* (Diptera: Asilidae)

**DOI:** 10.3390/toxins10110456

**Published:** 2018-11-05

**Authors:** Andrew A. Walker, James Dobson, Jiayi Jin, Samuel D. Robinson, Volker Herzig, Irina Vetter, Glenn F. King, Bryan G. Fry

**Affiliations:** 1 Institute for Molecular Bioscience, The University of Queensland, St Lucia, QLD 4072, Australia; jiayi.jin@uq.net.au (J.J.); s.robinson@imb.uq.edu.au (S.D.R.); v.herzig@imb.uq.edu.au (V.H.); i.vetter@imb.uq.edu.au (I.V.); 2 Venom Evolution Lab, School of Biological Sciences, The University of Queensland, St Lucia, QLD 4072, Australia; j.dobson@uq.edu.au; 3 Centre for Advanced Imaging, The University of Queensland, St Lucia, QLD 4072, Australia; 4 School of Pharmacy, The University of Queensland, Woolloongabba, QLD 4102, Australia

**Keywords:** venom, peptide, defensin, Asilidae, Diptera, insect venom, Asilidin, inhibitor cystine knot, extra-oral digestion

## Abstract

Assassin flies (Diptera: Asilidae) inject paralysing venom into insect prey during hunting, but their venoms are poorly characterised in comparison to those produced by spiders, scorpions, or hymenopteran insects. Here we investigated the composition of the venom of the giant Australian assassin fly *Dolopus genitalis* using a combination of insect microinjection assays, calcium imaging assays of mammalian sensory neurons, proteomics and transcriptomics. Injection of venom into blowflies (*Lucilia cuprina*) produced rapid contractile paralysis (PD_50_ at 1 min = 3.1 μg per fly) followed by death, and also caused immediate activation of mouse dorsal root ganglion neurons (at 50 ng/μL). These results are consistent with venom use for both prey capture and predator deterrence. Paragon searches of tandem mass spectra of venom against a translated thoracic gland RNA-Seq database identified 122 polypeptides present in the venom, including six linear and 21 disulfide-rich peptides. Some of these disulfide-rich peptides display sequence homology to peptide families independently recruited into other animal venoms, including inhibitor cystine knots, cystine-stabilised α/β defensins, Kazal peptides, and von Willebrand factors. Numerous enzymes are present in the venom, including 35 proteases of the S1 family, proteases of the S10, C1A, M12A, M14, and M17 families, and phosphatase, amylase, hydrolase, nuclease, and dehydrogenase-like proteins. These results highlight convergent molecular evolution between the assassin flies and other venomous animals, as well as the unique and rich molecular composition of assassin fly venom.

## 1. Introduction

Animal venoms display diverse pharmacologies that are adapted to the biology and ecology of the creatures that produce them. Studies on the venom systems of arachnids, snakes, and marine cone snails, among others, have revealed that venoms are typically complex composites of peptides, small molecules, pore-forming proteins, and enzymes that target the nervous and/or cardiovascular system to incapacitate prey or deter predators. Venom has evolved multiple times among animals [1,2,3], and the scope of venomous animal groups whose venom biochemistry has been examined is ever-expanding, having recently grown to encompass such animals as the platypus [4], centipedes [5], assassin bugs [6,7], and polychaete worms [8].

The assassin flies or robber flies (Asilidae) are a family of venomous dipteran insects that emerged in the Cretaceous era [9] and now comprise >7000 species with a worldwide distribution [10]. Assassin flies are sophisticated aerial predators, with flight and visual systems that are highly adapted to catch prey during flight [11]. Prey items include some aggressive and venomous species such as wasps, bees, dragonflies, and assassin bugs [12,13], and spiders taken from webs or whilst ballooning [14]. The potential for such prey to inflict damage on the fly, coupled with the possibility of prey escape, presents assassin flies with a need to rapidly immobilise a diverse range of prey.

Evidence that assassin flies use venom to immobilise prey was noted nearly a century ago by Whitfield [13], who compared the effects of bites inflicted by freely hunting asilids to mechanical damage inflicted with a needle. Whereas asilid bites caused rapid paralysis and death, the mechanical damage caused no effect or slow death over hours or days. Asilids inject venom through a robust, syringe-like hypopharynx that is connected to paired secretory glands in the thorax [15,16,17]. When injected into locusts, asilid thoracic gland extracts cause loss of balance, paralysis, antenna twitching, and eventual death, with an LD_50_ in large locusts of 0.004–0.5 gland equivalents per animal [17,18]. Sub-lethal doses result in paralysis that is reversible after hours or days. Mice injected intraperitoneally with thoracic gland extracts displayed reduced motor activity, hair bristling, muscle spasms, and respiratory arrest. Two to four gland equivalents of the asilid *Machimus rusticus* were sufficient to cause death of a mouse [17]. Together with the fact that asilids are reported to deliver a painful bite if handled by humans [19], these data are consistent with asilid venom containing toxins that rapidly paralyse prey during hunting, and cause pain in vertebrates during defensive envenomation.

Recently, the composition of the thoracic gland secretion of two species, *Machimus arthriticus* and *Eutolmus rufibarbis*, was investigated using transcriptomics and proteomics [16]. The venoms of both species were found to contain peptides with primary structural similarity to inhibitor cystine knots (ICKs) and von Willebrand factors, as well as peptides and larger proteins with uncharacterised structure and function (dubbed Asilidin families 1–10). One ICK-like peptide from *M. arthriticus*, U-Asilidin_1_-Mar1a, was synthesised and shown to have neurotoxic effects when introduced into the central nervous system of bees [16]. Although the thoracic glands have previously been proposed to produce enzymes that contribute to extra-oral digestion [17], enzymes were reported to make up only a minor fraction of the thoracic gland secretion of *M. arthriticus* and *E. rufibarbis* [16].

**Figure 1:**
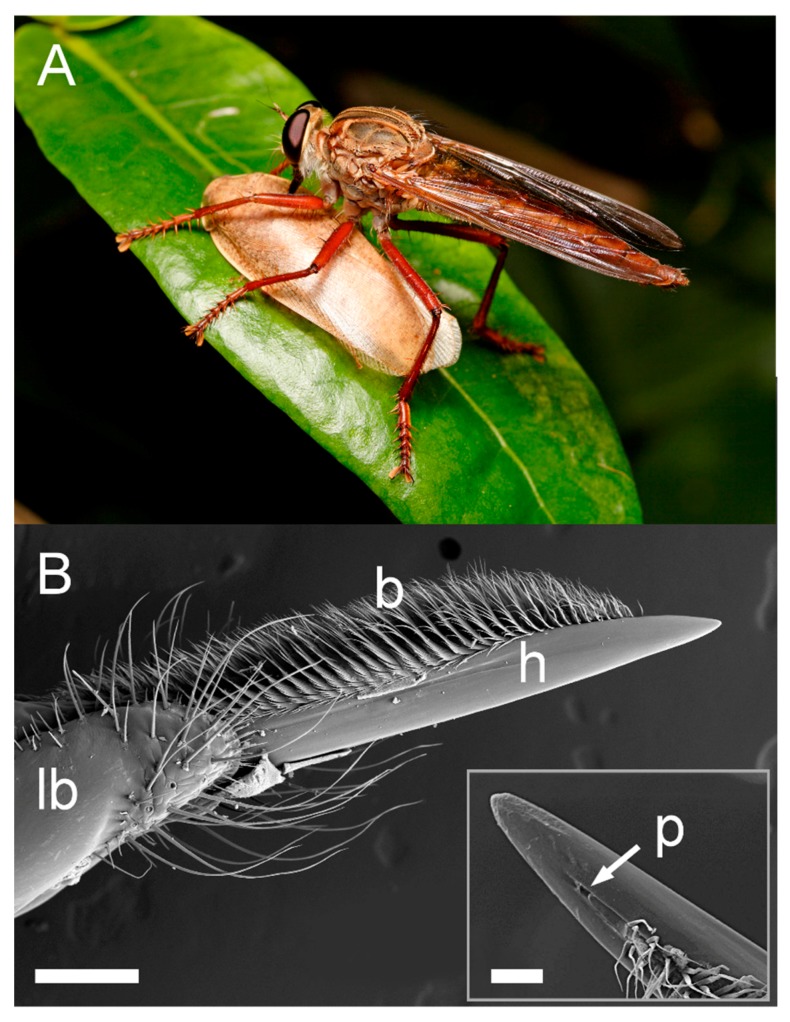
Giant assassin fly, *Dolopus genitalis*. (**A**) Female envenomating a cockroach (*Rhabdoblatta* sp.). Photograph: Alan Henderson, Minibeast Wildlife, Australia. (**B**) Scanning electron micrograph of hypopharynx adapted for venom delivery, lateral view. h, hypopharynx; lb, labium; b, hypopharyngeal bristles; scale bar, 200 μm. Inset: enlargement showing pore (p) near the tip that connects to thoracic gland duct. Scale bar, 25 μm.

Here, we have characterised venom harvested non-lethally from the mouthparts of the giant assassin fly *Dolopus genitalis* (Figure 1). The venom had marked effects on both insects (paralysis and death) and it rapidly activated mammalian nociceptors consistent with pain induction. Our method of venom collection also allows improved proteomic detection compared to previous studies, revealing *D. genitalis* venom as a composite of 123 peptides and proteins, including five families not previously detected in asilid venom.

## 2. Results

### 2.1. Bioactivity of Crude Venom

**Figure 2:**
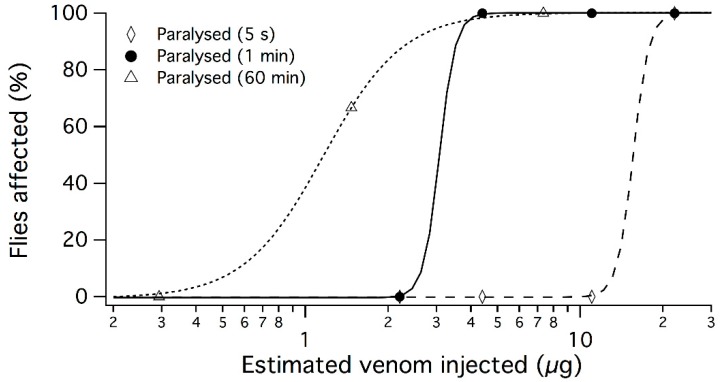
Dose-dependent paralysis of blow flies (*Lucilia cuprina*) after thoracic injection of whole *D. genitalis* venom.

Whereas electrostimulation (6–25 V) of head and thoracic regions produced no effect, gentle harassment of flies elicited a drop of venom from the proboscis, similar to results reported from some heteropteran insects [20]. Single harvests from individual flies collected 48–72 h previously using this method, yielded ~400 μg venom per fly. To investigate the biological effects of *D. genitalis* venom, whole venom was injected into blowflies (*Lucilia cuprina*). The behaviour of flies injected with 2 μL water was unaltered, whereas flies injected with 2 μL diluted venom (estimated concentration from A_280_ = 10.5 μg/μL) exhibited rapid contractile paralysis within 5 s, followed by death after several minutes (Supplementary Video S1). Lower doses of venom produced slower paralysis (Figure 2), similar to previously reported results using thoracic gland extracts [17,18]. The PD_50_ values for 60 min, 1 min and 5 s were, respectively, 1.16 μg, 3.07 μg, and 15.58 μg per fly (46.4, 122.8, and 623.2 μg/g). These results suggest that *D. genitalis* venom efficiently induces paralysis on the rapid time scale required to prevent prey escape, and that individual flies possessed >25 times the quantity of venom required to paralyse a blowfly within 5 s.

**Figure 3:**
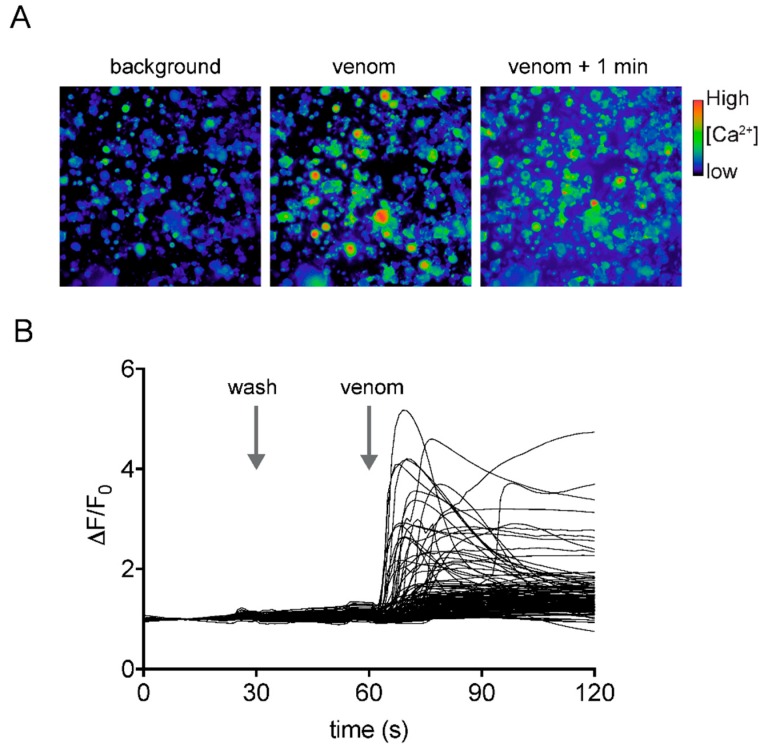
Effects of *D. genitalis* venom on mouse DRG neurons. (**A**) DRG cells before (background), directly after, and 1 min after addition of *D. genitalis* venom (50 ng/µL). Venom caused an immediate increase in intracellular calcium concentration in all cells followed shortly thereafter by release of dye from the cells into the extracellular medium. (**B**) Traces from all individual cells. Arrows indicate replacement of assay solution at 30 s (wash; negative control), and addition of venom at 60 s.

Application of diluted venom (50 ng/μL) to dissociated DRG neurons produced an immediate increase in intracellular calcium concentration (Figure 3). This effect was observed in all cells and was followed by release of the calcium-sensitive dye from the cells into the extracellular medium, suggestive of membrane disruption. However, we cannot rule out that additional, more specific effects, are masked by the dominant lytic effect. Nevertheless, these data are consistent with the ability of asilid venoms to cause pain during defensive envenomation of vertebrates [21].

**Figure 4:**
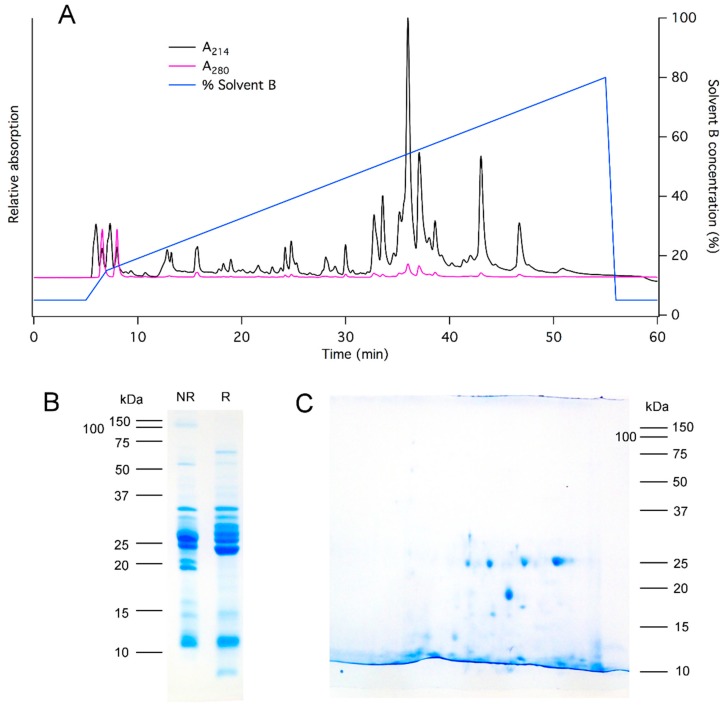
Composition of *D. genitalis* venom. (**A**) Chromatogram resulting from RP-HPLC fractionation of whole venom, consistent with the presence of numerous low molecular mass (1–10 kDa) peptide toxins. (**B**) 1D-SDS-PAGE separation of whole *D. genitalis* venom; molecular mass markers are on the left. NR, non-reduced; R, reduced. (**C**) 2D-SDS-PAGE separation of whole *D. genitalis* venom; molecular mass markers are shown on the right.

To investigate the mode of action of asilid venom toxins in more detail, we fractionated 1 mg whole venom using RP-HPLC (Figure 4A) and tested the effect of adding each reconstituted venom fraction to human neuroblastoma SH-SY5Y cells, using calcium imaging assays. These assays are capable of revealing modulation of human voltage-gated sodium channels Na_V_1.2, Na_V_1.3, and Na_V_1.7, voltage-gated calcium channels Ca_V_1.3 and Ca_V_2.2, and the α7 subtype of nicotinic acetylcholine receptor [22,23]. Assuming minimal losses during HPLC, the amount of each venom component tested (see Methods) is likely to be close to the quantity of that component in 100 μg venom, dissolved in 30 μL assay buffer (i.e., equivalent to the amount in a whole venom concentration of 3.3 μg/μL, much lower than the likely concentration of whole venom but much higher than the 50 ng/μL that showed potent activity on DRG neurons). However, we did not observe modulatory effects by any venom fraction, suggesting that the HPLC-fractionated venom components (likely to consist of small molecules, peptides, and small proteins) do not independently modulate these human ion channels, and the observed bioactivity of whole venom on DRG neurons is likely dependent on other mechanisms. For example, the calcium influx caused by whole venom could be due to the disruption of lipid membranes by larger proteins unsuited to isolation in their active form by HPLC. Unfortunately, difficulty obtaining sufficient animals for venom harvest precluded further bioassays, including the identification of insect-active toxins.

### 2.2. Proteomics Reveals Numerous Peptide and Protein Families in Asilid Venom

To examine the molecular mass profile of *D. genitalis* venom, we performed chromatography, electrophoresis, proteomics, and transcriptomic experiments. Fractionation of venom using a C4 reverse-phase column resulted in the elution of >12 peaks from the C4 column in the gradient range of 30–50% solvent B (90% acetonitrile, 0.043% TFA), suggesting the presence of numerous peptide toxins (Figure 4C). 1D and 2D SDS-PAGE revealed multiple protein bands and spots, with the most abundant in the range 10–35 kDa (Figure 4B,C).

To determine the primary structures of venom proteins and peptides, we combined RNA-Seq of *D. genitalis* thoracic glands with LC-MS/MS of venom obtained non-lethally from the mouthparts. Paragon searches of mass spectra collected from two samples of reduced, alkylated, and trypsinised venom, against a database of translated thoracic gland cDNA sequences, identified 123 amino acid sequences after filtering for quality (Supplementary Files S1 and S2). Based on sequence homology of identified proteins to known proteins, *D. genitalis* venom is rich in peptides (27 sequences), proteins of unknown function known only from asilid venom (24 sequences), S1 proteases (35 sequences), and other enzymes (21 sequences). Detected proteins were named according to rational nomenclature guidelines [24], with the prefix ‘Asilidin’ to remain consistent with a previous study [16]. Putative toxin families were assigned based on BLAST homology (and additionally cysteine scaffold patterns in the case of disulfide-rich peptides) and are indicated by a footnote in protein names. For example, the peptide U-Asilidin_12_-Dg3a has unknown function (indicated by the prefix U-) and belongs to Asilidin family 12. In the identifier ‘Dg3a’, Dg is an acronym for the binomial name of the species, the number is a unique identifier assigned to all peptides and proteins in the order they were discovered, and the final letter is used to distinguish subtypes with >90% identity in the amino acid sequence of the precursor.

We detected 27 peptides and proteins with homology to previously reported asilid venom proteins (Asilidin families 1–5, 8, and 9) [16]. Asilidin families 6, 7, and 10 were not detected in *D. genitalis* venom. The Asilidin-2 family (Supplementary File S3) is especially well-represented, with 13 members detected, each consisting of 229–365 residues including 4–7 cysteines in their predicted mature forms. Asilidin family 2 proteins are predicted to be rich in lysine (10–21% of putative mature residues) and α-helical structure (55–74% according to the secondary structure prediction algorithm GOR IV [25]), possibly suggesting a role in membrane disruption. This family is also abundant in venom, accounting for 25.3% of all precursor counts used for quantification. Transcripts encoding them are likewise abundant in thoracic gland mRNA, accounting for 22.1% of total FPKM units mapped to detected venom proteins. Another prominent family in *D. genitalis* venom, of which we detected 13 members, have been previously designated as ‘MBF2 domain proteins’ [16]. Here, we designate this family as Asilidin family 11 (Supplementary File S4); members of this family consist of 96–146 residues and all except U-Asilidin_11_-Dg15 are devoid of cysteine residues.

We detected a high number of putative S1 proteases (35) and other enzymes (21) in *D. genitalis* venom. In contrast, a previous proteomic study of asilid thoracic gland extracts found little evidence of enzymatic components, and the extract was reported to consist mostly of peptides and proteins of unknown function [16]. Examination of read counts and top precursor count intensities for enzymatic components detected (Supplementary File S1) suggests that enzymes detected in this study are unlikely to represent trace components or originate from tissue other than the thoracic glands. Transcripts encoding detected enzymes detected in the venom are well-expressed in the thoracic gland tissue (combined FPKM of 68,087 or 17.5% of total for detected proteins). The FPKM values for individual enzymes also correlate positively with their individual precursor count quantifications (*r* = 0.51, *p* < 10^−4^). Thus, the production of venom enzymes appears to be an important role of asilid thoracic glands.

### 2.3. Disulfide-Rich Venom Peptides

**Figure 5:**
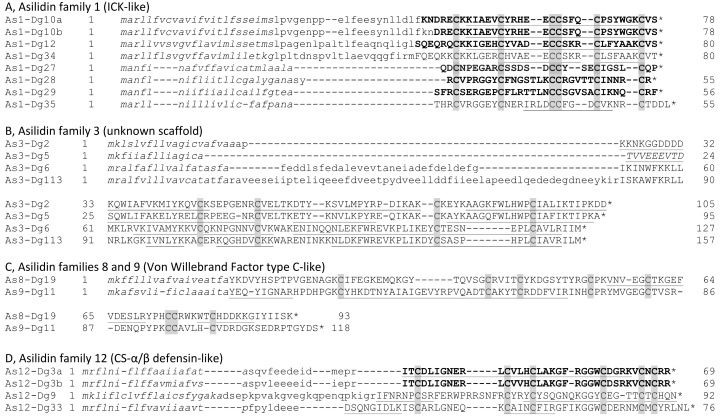
Amino acid sequence alignments of detected peptides in Asilidin families 1, 3, 8, 9, and 12. Whole peptides identified from reduced and alkylated (but not trypsinised) venom samples are shown in bold. Tryptic fragments detected in reduced, alkylated and trypsinised samples are underlined. Predicted secretion signal peptides are italicised, and cysteine residues in the mature sequence are highlighted in grey. Stars represent stop codons. The sequence shown in capital letters is the best estimate of the mature amino acid sequence of peptides present in venom based on all experiments.

Apart from Asilidin-11 family peptides and U-Asilidin_4_-Dg16, all other 20 detected peptides (those with <100 residues in the probable mature product) contain either 4, 6, or 8 cysteine residues that likely form intramolecular disulfide bridges, similar to as in the disulfide-rich peptides common in venoms of arachnids, cone snails and other venomous groups [26]. The primary structures of the detected peptides suggest the presence of several disulfide-rich peptide families known to occur in venoms of other animal groups (Figure 5). Asilidin-1 family peptides Dg10, Dg12, Dg34, and Dg35 show a cysteine scaffold similar to the inhibitor-cystine knot (ICK) toxins that are widely distributed in diverse venoms [27,28]. Asilidin-12 peptides Dg3, Dg9, and Dg32 are homologous to cystine-stabilised-α/β defensins (CS-α/β; HMMER *E* < 10^–5^ for Dg3 and Dg32). U-Asilidin_8_-Dg19 and U-Asilidin_9_-Dg11 are similar to type C single-domain von Willebrand factors (vWF; HMMER *E* < 10^−9^), and U-Asilidin_15_-Dg14 shows strong similarity to an isolated peritrophin-A domain (HMMER *E* < 10^−10^). The HMMER algorithm also detects similarity between asilid CS-α/β peptides and scorpion venom peptide toxins that modulate potassium channels (*E* = 0.0001 for U-Asilidin_12_-Dg3a) and between Asilidin-1 family peptides and conotoxins (*E* = 0.058 for U-Asilidin_1_-Dg12). However, the closest sequence homologues of Dg3a and Dg12 in GenBank’s nr database originate from Diptera (many predicted sequences with BLASTp *E* < 10^−5^ in each case), consistent with the convergent recruitment of venom peptides from non-neurotoxic venom peptide families.

**Figure 6:**
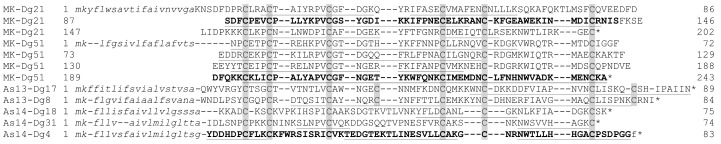
Amino acid sequences of multidomain Kazal (MK) proteins detected in *D. genitalis* venom (Dg21 and Dg51) together with putative Kazal-like peptides in families Asilidin-13 and Asilidin-14. Formatting as in Figure 5.

Asilidin families 13 and 14 contain peptides with mature sequences of 55–69 amino acid residues, with eight or six cysteine residues, respectively. Neither family has been previously reported in asilid venom. None of the five detected peptides in families 13 and 14 displayed homology to known proteins by BLAST search, nor contain domains recognised by HMMER searches of the Pfam database, and no homology is detectable between the Asilidin-13 and Asilidin-14 families. However, both families contain members with moderate homology to the multi-Kazal-domain-containing venom proteins Dg21 and Dg51 (lowest *E*-value hits, U-Asilidin_13_-Dg17 to Dg21 domain 1, *E* = 0.0003; and U-Asilidin_14_-Dg30 to Dg21-domain 3, *E* = 0.006). Alignment of Asilidin-13 and Asilidin-14 peptides with Dg21 and Dg51 (Figure 6) shows similar cysteine frameworks across these families, suggesting that Asilidin-13 and Asilidin-14 families may be derived from Kazal domains. However, tertiary structural information will be required to examine this hypothesis.

### 2.4. Mature Primary Structures of Peptides in Venom

To facilitate future functional characterisation of venom peptides, we investigated their mature primary structures in more detail by analysing untreated venom and reduced and alkylated (but not trypsinised) venom, using LC-MS/MS and MALDI-MS (Supplementary File S1 and Supplementary Table S1). After reconstruction of toxin masses using the ‘LCMS reconstruction’ algorithm in Analyst 6.1 software, the 50 most abundant masses within the range of 1–10 kDa were compared between untreated whole venom sample and the reduced and alkylated sample. We found 21 toxin masses that shifted by a mass corresponding to alkylation of six cysteines (17 peptides) or four cysteines (two peptides), or that were unchanged after reduction and alkylation (two peptides). Paragon searches of LC-MS/MS data from reduced and alkylated (but not trypsinised) venom resulted in the identification of seven peptides identified in shotgun proteomics experiments, as well as three additional Asilidin-1 family peptides, U-Asilidin_1_-Dg27–29 (Supplementary File S1). Peptides consisting of a single Kazal domain from the multidomain Kazal proteins Dg21 and Dg51 were also detected, suggesting that these multidomain proteins detected in trypsinised venom samples are actually precursors of peptides derived by proteolytic cleavage that are present in the mature venom (Figure 6).

We report the mature primary structures of 10 disulfide-rich peptides for which there is consistent evidence from LC-MS/MS and MALDI-MS of untrypsinised venom (Table 1). Of these peptides, the putative CS-α/β peptides U-Asilidin_12_-Dg3a and U-Asilidin_12_-Dg3b appear to be abundant in venom, accounting for 45.2% of precursor counts and 5.0% of FPKM values assigned to peptides in the non-trypsinised sample (Supplementary File S1). Asilidin-1 (ICK-like) peptides display the greatest diversity, with six peptides detected. Of these, U-Asilidin_1_-Dg10 is present as two subtypes that differ by two amino acids at their N-terminus. U-Asilidin_1_-Dg27 is a close homologue, differing by just seven amino acid substitutions, from U-Asilidin_1_-Mar1a from *M. arthriticus* that was shown to have neurotoxic activity when injected into the bee CNS [16].

## 3. Discussion

We examined the bioactivity and composition of venom of the giant assassin fly *D. genitalis*. The venom induced rapid paralysis and death when injected into blowflies, consistent with strong evolutionary selection pressure on asilid venom to rapidly incapacitate prey to facilitate capture during flight. The production of spastic paralysis has arisen convergently to other venomous lineages that hunt prey with high escape potential or ability to inflict damage, such as cone snails predating upon fish [29] and the long-glanded coral snake predating upon other venomous snakes [30]. When applied to mammalian DRG neurons, venom caused an immediate increase in intracellular calcium concentration, and this rapid activation of sensory neurons is consistent with the ability of the venom to cause pain in vertebrate predators. These results suggest that the secretion we analysed, obtained non-lethally from the mouthparts, is the same venom used by asilids in the natural environment to facilitate prey capture and ward off predators.

This study revealed the complexity of *D. genitalis* venom by combining RNA-Seq of *D. genitalis* thoracic glands with tandem mass spectrometry of venom to determine the primary structure of 123 venom peptides and proteins. These peptides and proteins show many similarities to those previously reported in asilid venom, including 27 peptides and proteins from Asilidin families 1–5, 8, and 9 [16]. However, in contrast to this previous study, we detected a larger number (123 from one species vs. 45 from two species) and a broader range of venom components, including additional venom peptide families, and a large number of putative enzymes that were previously thought to be rare in asilid venom [16]. Although species-specific differences may explain some of this discrepancy, we suggest that it is mainly due to differences in venom collection and processing, with the reduced sample processing in our study (i.e., venom collection directly from the mouthparts rather than by gland dissection followed by protein extraction and precipitation) allowing improved proteomic detection of venom constituents. This conclusion is supported by (1) the strong expression of enzyme-encoding transcripts we observed in *D. genitalis* thoracic glands and the positive and statistically significant correlation between FPKM values and precursor count quantifications for the individual detected enzymes; (2) the robust expression of S1 proteases observed in the thoracic glands in the study of Drukewitz and colleagues [16]; and (3) a previous finding that thoracic gland extracts (but not labial gland extracts) show strong proteolytic activity [17]. We cannot rule out the possibility that the secretion we analysed contains material originating from non-thoracic-gland tissue, such as the labial glands that also terminate at the mouthparts [15], but we find no evidence of this. The function of the labial glands, including if they function as part of the venom system, remains unknown. In any case, our results support the thoracic glands as the glandular source of both paralysing and pain-inducing venom, consistent with other studies [16,17,18].

Overall, asilid venom is generally similar in composition and bioactivity to other groups of insects that use salivary venoms for hunting and defending themselves from predators, such as assassin bugs [6,7] and other heteropteran insects [31]. Most likely, these similarities in composition reflect convergent evolution of venom to facilitate both prey capture and extra-oral digestion. In contrast, arachnids, which use venom for prey capture and defence, and a separate secretion for extra-oral digestion [32], feature venoms consisting mostly of peptides [33,34]. The dual role of asilid venom may also explain the relatively high PD_50_ values of *D. genitalis* venom obtained compared to those reported for non-salivary insect venoms (e.g., [35]). In any case, individual assassin flies yielded ~400 μg per harvest, >25 times the quantity of venom required to induce paralysis of a blowfly within 5 s (15.6 μg), suggesting *D. genitalis* venom is well-adapted to facilitate prey capture in the context of how it is used in nature.

Like other insect venoms, *D. genitalis* venom contains a wide range of peptides with diverse structures. Primary structural characteristics suggest the presence of CS-α/β defensins (Asilidin family 12), ICK toxins (Asilidin family 1), vWFs (Asilidin families 8 and 9), Kazal domain peptides (derived from precursors Dg21 and Dg51 and possibly also Asilidin families 13 and 14), and a peritrophin domain peptide (U-Asilidin_15_-Dg14). Although 3D structural studies are required to test these predictions, these results suggest that multiple peptide families have been recruited into venom convergently in asilids and other venomous animals. CS-α/β defensins and ICK toxins dominate the venoms of scorpions and spiders, respectively [36,37]. Toxins with vWF domains occur in some scorpion venoms [37,38], Kazal domain peptides are known from venoms of snakes and heteropteran insects [7,39], and a peritrophin domain peptide has been reported in venom of the wasp *Pteromalus puparum* [40]. Peptides without disulfide bonds (Asilidin families 4 and 11) also occur in asilid venom, as they do in the venoms of hymenopterans and arachnids [34,37,41]. In addition, some asilid venom peptides show disulfide scaffolds of unknown structure that may represent novel folds, such as the four-cysteine-containing Asilidin family 3 peptides. These results suggest that assassin flies have recruited venom proteins from diverse genetic resources in a manner convergent with other venomous animals, by selecting peptides with particular physicochemical properties [26].

The mechanisms of action of *D. genitalis* venom peptides are unknown, but similar peptides in other animal venoms act as ion channel modulators and membrane disruptors [42,43]. However, we did not detect peptide modulation of a range of human ion channels tested (Na_V_1.2, Na_V_1.3, Na_V_1.7, Ca_V_1.3, Ca_V_2.2, and α7 nicotinic receptors). Possibly, *D. genitalis* venom peptides act selectively on insect ion channels, or function by disrupting lipid bilayers or other targets. U-Asilidin_1_-Mar1a from *M. arthriticus* has been shown to have neurotoxic effects when introduced into the ocelli of bees, and hence probably acts on an unknown ion channel or receptor in the insect CNS [16]. The almost instantaneous activity of *D. genitalis* whole venom when injected into fly abdomens suggests it contains components that act on peripheral targets in insects, such as at neuromuscular junctions.

The function and pharmacology of the vast majority of venom components of asilids awaits further study. The bioactivity and composition of assassin fly venom documented in this study will assist further efforts to characterise the structure and function of venom toxins, enriching our understanding of the evolution of venom use in Asilidae. In addition, the high diversity of structurally distinct peptide families in asilid venom makes Asilidae an interesting prospect for the discovery of novel peptides for use as pharmacological tools, therapeutic leads, and bioinsecticides [44,45,46].

## 4. Materials and Methods

### 4.1. Insects and Venom Collection

Giant assassin flies (*Dolopus genitalis*) were purchased from Minibeast Wildlife, QLD, Australia. To harvest venom, flies were first anaesthetised with CO_2_ and then restrained by holding the wings. As the flies regained alertness, gentle harassment elicited venom from the end of the proboscis, from where it was transferred using a pipette tip containing a small volume (~10 µL) of water to an Eppendorf tube and stored at −20 or −80 °C. Venom collected in this way was diluted approximately 10-fold in ultrapure water. For this reason, the protein concentration of diluted venom solutions in this study was estimated using their absorbance values at 280 nm as measured using a Nanodrop spectrophotometer.

### 4.2. Toxicity Assays

Newly emerged sheep blowflies (*Lucilia cuprina*) were injected with 2.1 μL diluted whole venom or ultrapure water using a hand microapplicator (Burkard Scientific, Uxbridge, Middx, UK) that allows injection of precise small volumes of liquid. The proportion of paralysed flies was recorded at 5 s, 1 min, and 60 min.

### 4.3. Calcium Imaging of Mouse Sensory Neurons

Dorsal root ganglia (DRG) from 4 week old male C57BL/6 mice were dissociated and plated in DMEM (Invitrogen, Carlsbad, CA, USA) containing 10% foetal bovine serum (FBS) (Assaymatrix, Ivanhoe North, VIC, Australia) and penicillin/streptomycin (Gibco, MD, USA) on a 96-well poly-D-lysine-coated culture plate (Corning, ME, USA) and maintained overnight. Cells were loaded with Fluo-4 AM calcium indicator according to the manufacturer’s instructions (ThermoFisher Scientific, Waltham, MA, USA). After loading (30 min at 37 °C then 30 min at 23 °C), the dye-containing solution was replaced with assay solution (Hanks’ balanced salt solution, 20 mM HEPES). Fluorescence corresponding to [Ca^2+^]*_i_* of 100–150 DRG cells was monitored in parallel using an Nikon Ti-E deconvolution inverted microscope, equipped with a Lumencor Spectra LED lightsource. Images were acquired at 20× objective at one frame per second (excitation 485 nm, emission 521 nm). Baseline fluorescence was monitored for 30 s. At 30 s, assay solution was replaced with assay solution (negative control), and at 60 s with *D. genitalis* venom (50 ng/µL in assay solution). Experiments involving the use of mouse tissue were approved by The University of Queensland animal ethics committee (TRI/IMB/093/17).

### 4.4. Calcium Imaging of Human Neuroblastoma SH-SY5Y Cells

To investigate the activity of venom fractions on human ion channels, human neuroblastoma-derived SH-SY5Y cells (ECACC, Salisbury, Wiltshire, UK) were maintained at 37 °C/5% CO_2_ in RPMI medium containing 15% foetal bovine serum (FBS) and 2 mM l-glutamine. Cells were plated in 384-well black-walled imaging plates (Sigma Aldrich, St. Loius, MO, USA) at a density of 50,000 cells/well and cultured in growth medium for 48 h. Dye-loading with Calcium 4 No-Wash dye (Molecular Devices, San Jose, CA, USA) was achieved by incubation with dye solution, prepared in physiological salt solution (PSS; 140 mM NaCl, 11.5 mM glucose, 5.9 mM KCl, 1.4 mM MgCl_2_, 1.2 mM NaH_2_PO_4_, 5 mM NaHCO_3_, 1.8 mM CaCl_2_, 10 mM HEPES, pH 7.4) according to the manufacturer’s recommendations for 30 min at 37 °C. Lyophilised venom fractions were resuspended in 30 μL PSS, of which 3 μL was added to dye-loaded cells using a FLIPR^TETRA^ (Molecular Devices) plate reader with a final assay volume of 30 μL. Real-time fluorescence responses (excitation, 470–495 nm; emission, 515–575 nm) were measured for 5 min (300 reads).

### 4.5. Chromatography

For reverse-phase high performance liquid chromatography (RP-HPLC), 1 mg of whole venom (A_280_ equivalent) was diluted five-fold in loading buffer consisting of 95% solvent A (0.05% TFA) and 5% solvent B (0.043% TFA, 90% acetonitrile). After centrifugation (10 min, 17,000 rcf, 4 °C), the supernatant was loaded on a C4 Jupiter column (100 μm particle size, 300 Å pore size, Phenomenex #00G-4168-NO) and fractionated using a gradient of 5–80% solvent B.

### 4.6. Electrophoresis

For 1D electrophoresis, 25 μg of venom with sample loading buffer (with or without 50 mM dithiothreitol, DTT) was denatured (100 °C for 4 min) and electrophoresed on a 12% polyacrylamide gel at 90 V for 90 min before staining with Coomassie Brilliant Blue G250. For 2D electrophoresis, 400 μg freeze-dried venom was dissolved in 125 μL solubilisation buffer (8 M urea, 4% CHAPS, 110 mM DTT, 0.5% ampholytes: BioLyte^®^ pH 3/10, Bio-Rad #1631112) and 0.01% bromophenol blue. Isoelectric focusing (IEF) was performed overnight on ReadyStrip non-linear pH 3–10, 7 cm IEF strips (#1632002, Bio-Rad, Hercules, CA, USA) using a PROTEAN^®^ i12^TM^ IEF system (Bio-Rad). IEF conditions were 100 V for 1 h, 500 V for 1 h, 1000 V for 1 h and then 8000 V, for a total of 98,400 V/h, 50 μA current per strip at 20 °C. Strips were equilibrated for 15 min in EQ buffer (50 mM Tris–HCl, pH 8. 8, 6 M urea, 2% SDS, 30% glycerol) + 2% DTT, followed by 20 min alkylation in EQ buffer + 2.5% iodoacetamide. Immobilised pH gradient (IPG) strips were then imbedded into a 12% resolving gel, enclosed in 0.5% agarose, and second dimension electrophoresis performed at 20 mA per gel for 60 min before staining with Coomassie Brilliant Blue.

### 4.7. Transcriptomics

Four male flies were sacrificed by gassing with CO_2_ for 10 min. The paired venom glands were dissected from the thorax, cleaned in phosphate buffered saline (PBS), and immediately frozen in liquid nitrogen. Poly-A(+) RNA was isolated using a Dynabeads mRNA Direct Kit**™** (Invitrogen, Carlsbad, CA, USA) according to the manufacturer’s instructions, yielding >5 μg mRNA. Library preparation and sequencing was performed by the Australian Genome Research Facility on an Illumina NextSeq instrument, yielding 21,250,270 adaptor trimmed 150 bp paired-end reads. Reads were *de novo* assembled into contigs using Trinity 2.4.0 and CLC Genomics Workbench. For the Trinity assembly, default trimming settings and a minimum contig size of 120 bp yielded 83,500 contigs. For CLC Genomics Workbench, reads were trimmed with a threshold parameter of 0.005, and four assemblies (minimum contig size 120 bp) were produced using word (k-mer) parameters of 31 (44,176 contigs), 42 (47,707 contigs), 53 (51,439 contigs), and 64 (61,120 contigs). From these 287,942 contigs, 429,155 translated ORFs >50 bp were extracted using TransDecoder 5.3.0 and added to a library of common tandem mass spectrometry contaminants (MS/MS) for use in proteomic experiments.

### 4.8. Mass Spectrometry

For matrix-assisted laser desorption/ionisation time-of-flight (MALDI-TOF) MS, venom was diluted in MALDI solvent (70% acetonitrile, 1% formic acid) and spotted together with the same volume of α-cyano-4-hydroxycinnamic acid (5 mg/mL in MALDI solvent). Spots were analysed on a SCIEX MALDI-TOF 4700 mass spectrometer, with a laser power of 3400–3800 V (reflectron mode) or 4000–4200 V (linear mode).

For liquid chromatography-tandem MS (LC-MS/MS), venom was centrifuged (10 min, 12,000 rcf, 4 °C) to remove particulate matter, and 5–50 µg of clarified venom was incubated with 20 µL reduction/alkylation buffer (50 mM ammonium carbonate pH 11.0, 1% iodoethanol, 0.025% triethylphosphine in 48.5% acetonitrile) for 2 h at 37 °C. The reduced and alkylated sample was then lyophilised, resuspended in 10 µL digestion reagent (20 ng/µL proteomics grade trypsin Sigma #T7575, in 40 mM ammonium bicarbonate pH 8.0, 5% acetonitrile) for 16 h at 37 °C. The reaction was then terminated by addition of 20 µL 5% formic acid and the tryptic digest was lyophilised. Digests were resuspended in 1% formic acid, 2.5% acetonitrile and loaded onto a 150 × 0.1 mm Zorbax 300SB-C18 column (3.5 µm particle size, 300 Å pore size, Agilent catalog no. 5065-9910) on a Shimadzu Nano LC system. The LC outflow was coupled to a SCIEX 5600 Triple TOF mass spectrometer equipped with a Turbo V ion source. Peptides were eluted over a 70 min gradient of 1–40% solvent B (90% acetonitrile, 0.1% formic acid) in solvent A (0.1% formic acid) at a flow rate of 0.2 mL/min. MS1 scans were collected between 350 and 1800 *m*/*z*, and precursor ions in the range *m*/*z* 350–1500 with charge +2 to +5 and signal >100 counts/s were selected for analysis, excluding isotopes within 2 Da. MS/MS scans were acquired at with an accumulation time of 250 ms and a cycle time of 4 s. The “rolling collision energy” option was selected, allowing collision energy to be varied dynamically based on *m*/*z* and *z* of the precursor ion. Up to 20 similar MS/MS spectra were pooled from precursor ions differing by less than 0.1 Da. The resulting mass spectra in WIFF format were then compared with a library of translated ORFs extracted from transcriptomes generated from RNA-Seq experiments (together with a list of common MS contaminants) using a Paragon 4.0.0.0 algorithm implemented in ProteinPilot 4.0.8085 software (SCIEX). A mass tolerance of 50 mDa was used for both precursor and MS/MS ions. For reduced, alkylated, and trypsinised samples, the protein identification threshold was set at three detected tryptic fragments each with >95% confidence if no secretion signal peptide was predicted by SignalP 4.1, or two detected tryptic fragments if a signal peptide was predicted. Tandem mass spectra were also analysed manually using Analyst 6.1 and PeakView software (SCIEX). Identified amino acid sequences and cDNA sequences were uploaded to GenBank and were allocated the accession numbers MK075118–MK075240.

## Figures and Tables

**Table 1:** Mature peptides detected in *D. genitalis* venom.

Peptide	Amino Acid Sequence	Amino Acids	Putative Fold	LC-MS/MS Monoisotopic Mass, Untreated (Da)		LC-MS/MS Monoisotopic Mass, after RA^a^ (Da)		LC-MS/MS Abundance Rank ^b^	MALDI Mono-Isotopic Mass ^b^	MALDI Peak Signal Rank	Transcript Expression (FPKM)
				Predicted	Measured	Predicted	Measured				
**U-Asilidin_12_-Dg3a**	ITCDLIGNERLCVLHCLAKGFRGGWCDGRKVCNCRR	36	CS-α/β defensin	4057.97	4057.87	4328.15	4328.04	1	4057.85	4	5439
**U-Asilidin_12_-Dg3b**	ITCDLIGNERLCVVHCLAKGFRGGWCDSRKVCNCRR	36	CS-α/β defensin	4073.97	4073.85	4344.15	4344.03	2	4073.85	7	2558
**U-Asilidin_14_-Dg4**	YDDHDPCFLKCKFWRSISRICVKTEDGTEKTLINESVLLCAKGCNRNWTLLHHGACPSDPGG	62	Kazal?	6970.31	6970.17	7240.49	7241.37	25	nd	nd	26,171
**U-Asilidin_1_-Dg10a**	KNDRECKKIAEVCYRHEECCSFQCPSYWGKCVS	33	ICK	3921.70	3921.57	4191.88	4191.77	78	3921.5	9	2721
**U-Asilidin_1_-Dg10b**	DRECKKIAEVCYRHEECCSFQCPSYWGKCVS	31	ICK	3679.56	3679.45	3949.74	3949.64	11	3679.5	2	2721
**U-Asilidin_1_-Dg12**	SQEQRQCKKIGEHCYVADECCSKRCLFYAAKCVS	34	ICK	3906.76	3906.64	4176.94	4176.83	28	3906.6	3	2507
**U-Asilidin_15_-Dg14a**	RECPTVENEKDIAVHLPHKDCSKYYACVKGKKIERKCPRGLLFNKTLQVCDFPERVKC	58	Peritrophin domain	6755.44	6755.28	7025.62	7026.48	26	nd	nd	1303
**Kazal protein Dg21 domain 2**	SDFCPEVCPLLYKPVCGSYGDIKKIFPNECELKRANCKFGEAWEKINMDICRNIS	55	Kazal	6307.00	6306.9	6577.18	6577.09	24	nd	nd	2172
**U-Asilidin_1_-Dg27**	pyroQ-DCNPEGARCSSDSDCCYSECIGSLCQP	27	ICK	2946.03	2945.94	3216.21	nd	116	2945.9	11	4377
**U-Asilidin_1_-Dg28**	RCVPRGGYCFNGSTLKCCRGVTTCINNRCR	30	ICK	3330.54	3330.42	3600.72	3601.63	464	nd	nd	93
**U-Asilidin_1_-Dg29**	SFRCSERGEPCFLRTTLNCCSGVSACIKNQCRF	33	ICK	3708.67	3709.56	3978.85	3978.74	51	nd	nd	1827
**Kazal protein Dg51 domain 4**	DFQKKCKLICPALYAPVCGFNGETYKWFQNKCIMEMDNCLFNHNWVADKMENCKA	55	Kazal	6472.94	6473.8	6743.12	6742.94	31	nd	nd	2339

^a^ RA, reduction and alkylation (with iodoethanol) ^b^ From untreated venom.

## Supplementary Materials

The following are available online at http://www.mdpi.com/2072-6651/10/11/456/s1, Supplementary File S1: XLSX spreadsheet containing detailed sequence, annotation, and mass spectrometric identification statistics for all peptides and proteins identified in this study; Supplementary File S2: FASTA file containing nucleotide and amino acid sequences identified in this study; Supplementary File S3: Alignment of detected protein sequences that belong to the Asilidin-2 family; Supplementary File S4: Alignment of detected protein sequences that belong to the Asilidin-11 family; Supplementary Table S1: Masses detected using MALDI-MS; Supplementary Video S1: Rapid paralysis of blowflies (*Lucinia cuprina*) after injection of ~15.6 μg (estimated from A_280_) whole venom.
