# Supplementary material for: Buzz Kill: Function and Proteomic Composition of Venom from the Giant Assassin Fly Dolopus genitalis (Diptera: Asilidae)
Source: Toxins (Basel). 2018 Nov 5;10(11):456. doi: 10.3390/toxins10110456 (PMC6266666; doi:10.3390/toxins10110456)
Supplement: Supplementary file 1 [file toxins-10-00456-s001.zip › Supplementary File 3.docx]

Dg101 1 *mkllvtilistllvqgitp----*YPE---PSFGDMMKGIAEG---------------------------------------------------VGKIGTV 42

Dg46 1 *mkilvaaiicaflcqgvls*NPVPEPD---PFFGEIAQKIADG---------------------------------------------------ISGAASS 46

Dg104 1 *mkilatifvslllcqaatp----*MP-----IFGSIIGSITGG---------------------------------------------------ITKVVDK 40

Dg40 1 *mrftlailcallavnfsss----*APWDVGGWFKDAGKWIEGAAKDTGKWVEGAAKDTGNWVEGAAKDTGNWVEGAAKDTDRWVKGAAKDTGKWVEGAAKD 96

Dg47 1 *mkltigflllvvvlqcmttpaes-------------*KSLQ------------------------------------------------------------ 27

Dg48 1 *mrfalffiaafvllqcipqses-------------*KNWLSKR---------------------------------------------------------- 29

Dg70 1 *mrffvvillaavtaqtvlg----*KP----GIIGDITSSIK------------------------------------------------------------ 32

Dg83 1 *mrlllvlliasigtqstla----*EP----GWWGDILSSVK------------------------------------------------------------ 32

Dg115 1 *mriafflllalcsysaka----*QTEN---DLDVAYLEDIQ------------------------------------------------------------ 33

Dg37 1 *mkfaifvllglcaivaha----*SYE------LPEYDDDFE------------------------------------------------------------ 30

Dg38 1 *mkfailaflglcavvaa------*YE-------VEYDFEID------------------------------------------------------------ 27

Dg56 1 *mksfilvllglcaiasa------*LE------PVEFGVDVE------------------------------------------------------------ 28

Dg81 1 *mkfelfiflisaifvakg*-QEATYS--------------------------------------------------------------------------- 24

Dg101 43 IGGIVSGVVEGAGKIVIGV------------------INTTQGLVNNVLNATVAIVSGVAKEVANVTGGILQRVADLSQATEDAFRRMIRIGDGKIKDAA 124

Dg46 47 AGNIVSKIANGWGKMANGM------------------IDFGRSVAESAINGSVNIVNKSANEWARMTGLVAGKFANMTRSVEDSVKNIFNKGSNSFKKIG 128

Dg104 41 TADIISGTVNKVGSVTNGI------------------VGKISDVTKGAINVGVDFVNKVAGGMASITGATAGKFANITASIENSIKNAMGIGGNTFKKIS 122

Dg40 97 TGKWVEGAAKDTDRWVKGAVKDTEKWVKGAWRDVTDFFDKAGAEITKAANAVKNEWNKFEKNASEFFKNA---QAEVKKAIEDIKKRIDETGESIVKVIY 193

Dg47 28 --------------------------------------KKASEVFNKAVNKIKEAVNKLKTEAKAFFQKA---KEGIKKGLSAMKEKLKTLPDKAMRAVF 86

Dg48 30 -------------------------------------TNKAAEVFKKAVDKIKQKIGFVKNECKAFFKKV---KDGIKKSVAKIKSEVEKTGDSAMKVIF 89

Dg70 33 --------------------------------------DASKGIWDKIAEKAGAAIDKAIEKTKDMYKWALGEFAAMGDKLKDMEKKMIASGSDMLRSSF 94

Dg83 33 -----------------------------------------------------DSLAKIAEKAQDLYKKA-KYFPILDEAIKQLEKDLAKSG---VITSL 75

Dg115 34 -------------------------------------MQVRGPILDAALD--------------------------IVKKINEEVRKA-------IEAII 63

Dg37 31 -------------------------------------IEPQNKITDE-----------------------------IRAKIEELIKRASAE----FGKIS 60

Dg38 28 -------------------------------------IEPQNKVIDS-----------------------------IKAKIEELLKRATDD----FSIVA 57

Dg56 29 -------------------------------------FEPKNELPDI-----------------------------IRNKIEEFLKKVIED----FDTIT 58

Dg81 25 ----------------------------------------------------------------------------LSEATKLHLLKLVEG----VTFMS 44

Dg101 125 VAIKERVGEINGKVKDMMGEVDKKFEEGMKIVNASIEESSAKLTDMVNEIKEKYKDAKDLSKEDSEKIEQLLKDFVTSSSQNLSECAKEIVEPMKSLHEE 224

Dg46 129 EEIKSRVSEIREQVEGMLAEIDTKFKEGIEKVNATLKENKQKIEEIVSEIKEKLQNQKDVSKEDMEKIDKLIDDFIKESDELLSACAEQIVKPIKDMYKD 228

Dg104 123 EEVQKRMNSVRDKVTSAMKEAEDKYNEGVAKLNDTMLQNTKKIEEIASEIRNKY---KDVTEDSKKKIDDLINEFVYNSINECQQCAKEILVPIQGMFNN 219

Dg40 194 DKIYEWLQAARAKLEDYSHVADEKFEKAMEKVRQKLFTATSQIEGFLNRMKE-----IINKKTDQKKGLEILEKYTLESTEKIESCADAAIDPIRDLYEA 288

Dg47 87 QGLHDAIEKARKKLKETNEVADEKFYKAMKKAHDKFFDVTSQIEKFVTKQKE-----AITKKQEKEKATEILDDFVIRSSEKIESCSDAALGPVKDLYER 181

Dg48 90 KQIYNGLDKARKKLDDVSEIADKKFYSGMEKAHNLFFTVTSQIEKFVEKQKK-----IVEKKKDKDTGLKIIDDFVIKSSGKVESCSDAALNPVKELYEK 184

Dg70 95 DAAKRFVNSSEEQIQDMLKIIDEKLELATKESVKIVLKETSDLKKWANRTLE-----GVNDPAKRAQAEKIVGKFVDKNLEKMNKCSQEATKPIKEVYEY 189

Dg83 76 EMARKFVDTLEIQVQDLLRLVDEKLEAISIFIAKALLKEASGLNKWANKTLE-----KFDDPVKRAKAEKIVEKFMDKSIQKISRCARDVAKPIHDLYGM 170

Dg115 64 AKTEEIIKDVREKIENAAKTAMDKIRALVKKIEERLAKIAKK---------------GDVAKSCVDKGMPKIKEAIQNAETDNKQCIWSTLEQAKNIVEE 148

Dg37 61 DKVSEIMSRVREHARKIIMKAKDAVDKAKDDIEGKLDELKKK---------------GGAFAECAKKLRPEYRQVEQETMTGLKKCAGDAMISNEEHRQE 145

Dg38 58 DKAKGIIGKLNDAAADLVLKAKGKANSIKDEIEGKLDDMKKK---------------GGEVAACAKRLEPDYWQVERDAMADVKVCAGDVYVAANDMQDQ 142

Dg56 59 TKIENMVVRLNGQIAELVAMAKDKLHAVKTEVQGKLAEVKKQ---------------GGEAAVCATKLEPEYHKVEEDFLDEIKTCAAEARRATHQIRED 143

Dg81 45 ERAAEILQNVEFQMKSFNSSANMNLTEVHTKISKVLDDVSSN----------------KEYKKCIDRERMQLRFILQKARADVNQCLRAGIIRGQVLRNN 128

Dg101 225 AQTAINNILPVVEEAIKNLNQCLKDNRFKPTKIVECAKEAQSNL-PTIEHMEEFAKKLKAEIDNFKLDDILDM-SCIEENLNRIKTRKEELEKEIAKIVD 322

Dg46 229 AQNNINGVLPVVEDAIKDLGDCLKDNMLNPVKMTQCAMEAQKHL-PTIASMGDFVKNLKKQIDEANFDPMSNM-KCMKDSLEKIKENKDKLDAEISKILE 326

Dg104 220 TLDAINQILPAIEGVAVSMEECMKQKSISQTKLIKCAMDAKKKI-PSLGTMDEFVSNLKKELDKYKFDDLGK--TCMGPTLEKIMKHKEDLEKKIEEIVS 316

Dg40 289 SKDFIRSAIQRLDDVIRDMGACLDKG--VSKAAFNCGKDLKKSAKAALKELGSELISMKATLISSAISEAFNF-VCIGKTRAEVEFAKANIEAQIKALK- 384

Dg47 182 TKAVINGSVDKMENILKASRECMD----AVSKIVKCAPEIARNAKKQLSDLGNELLELKSKLLGVAMNEVTNF-ICIGRTRTDVELDKMSVKSRIDDIK- 275

Dg48 185 TKAIIKGSIDKVEEIIRQTEKCMDMG--MKTETLLCARNIGSIATEQLYKMGEEIMELKNKLIGGAVKELLNF-VCVGKTRSGVEIDKLTVEKKIKNLK- 280

Dg70 190 ANATAAEAAAVARKIVNMLEICYK-----ATNIMTCAIELPQVIKDGNDLMITKFAPMKAKIAAIGTKGALKFSSCVATAKIAMEWEKASIENKINDL-- 282

Dg83 171 ANIIAMEATKVARNAVNTLDTCSK-----GNDIKDCFKKLPAILEEGRNLIYAKISPMKSQLKSIASGTSISLSSCIATAKITIEINKSAVESRLKSLN- 264

Dg115 149 IKKHIEDIQDKCDANKDLVAECMETNG-SVWGATKCITKQVWTFNKNIFGMLGDVKNLLFEAVKSSFQVVANGISCLTDVGRTAEKQIIGAVKGIEQCIA 247

Dg37 146 VGKAVAAVRVNLREIRDQVADCLGWN---PIKTGKCIFEKTSELKKLVDGVVSASRDALRLTNKRAREIVTKVKACNSGVLNMASQRVKELNEKLSDCYK 242

Dg38 143 VGRGMSLISSKIREFRDMVGDCLGWN---PISATKCVYDKFDDLKKIMGGIMEAAKDALSAAQHKSVEIMNEAHQCNAKALSKASMRIKELNQKLANCYN 239

Dg56 144 VLKGIANVKNHVKDFREMLKDCLKWN---PIRFVKCMKEKHDEFKAVIDDIFNSAQGALRIAQMKAVQISGEAHVCNVYAVKSAEAKIEKLNEKLSSCGK 240

Dg81 129 TIQPLLGIR-----LNHFIQKCF------VHEKNECTEQEAAVVKEEAQLAIKMAYDNLYKAGGELKPLANKVLQCLRNIDERTKSQAKSFLDATMKRNA 217

Dg101 323 KKE------------------------------------------------------------------------------------------------- 325

Dg46 327 KKEE------------------------------------------------------------------------------------------------ 330

Dg104 317 KE-------------------------------------------------------------------------------------------------- 318

Dg40 385 ---------------------------------------------------------------------------------------------------- 384

Dg47 276 ---------------------------------------------------------------------------------------------------- 275

Dg48 281 ---------------------------------------------------------------------------------------------------- 280

Dg70 283 ---------------------------------------------------------------------------------------------------- 282

Dg83 265 ---------------------------------------------------------------------------------------------------- 264

Dg115 248 SNKLEDFNNRIMSY-------------------------------------------------------------------------------------- 261

Dg37 243 NAGMFY---------------------------------------------------------------------------------------------- 248

Dg38 240 ADILLY---------------------------------------------------------------------------------------------- 245

Dg56 241 FEMFVF---------------------------------------------------------------------------------------------- 246

Dg81 218 RQQLN----------------------------------------------------------------------------------------------- 222
