# Supplementary material for: Buzz Kill: Function and Proteomic Composition of Venom from the Giant Assassin Fly Dolopus genitalis (Diptera: Asilidae)
Source: Toxins (Basel). 2018 Nov 5;10(11):456. doi: 10.3390/toxins10110456 (PMC6266666; doi:10.3390/toxins10110456)
Supplement: Supplementary file 1 [file toxins-10-00456-s001.zip › Supplementary File 4.docx]

As11-Dg1 1 *mksf---------gvlallviaacva---------------------------------------------esya*QSHNVVFGSIQPGDRKLFQQIVMKK 46

As11-Dg13 1 *mkyf---------gilalalva-cva---------------------------------------------vsna*QSNNMSWGSIGPGDSLLDRQIISKP 45

As11-Dg7 1 *mkyf---------gilalalaa-cvv---------------------------------------------itna*QSHNSLWGSIGPGDNLLDRQIIKKS 45

As11-Dg32 1 *mkyf---------mvfafaiia-cls-----------------------------------------------avsa*SNSTFGAISPSDSKLHEENVFKS 43

As11-Dg36 1 *mnnf---------vtvllmvig-slv---------------------------------------------nnvys*QNNIIWGAVGPNDVHLFRERVIEP 45

As11-Dg15 1 *mkmrhl-------ivvilavigicff---------------------------------------------svsa*QKHSVTFGKAGPEDRLLYSTLVKRP 48

As11-Dg20 1 *marf--------ialaclllvaicaa*S---PEFE--------SSIEVIDNIDTYLADNPDAELIALQVQ--SMPFAKSRYSFGRRVPGDRILAKRNENSN 79

As11-Dg23 1 *marf--------ialacvllaa-cava*SEFDQFD--------STVEVVDNINTYLVDNPDAELIELQAQ--SMPFAKTRYTFGQRVNGDRVLAQRNDNFN 81

As11-Dg22 1 *msrf-------fitlaciafvgvcsa*----QNLD--------QHVQFVEDIEKFRAENPEVVLTPLTVE-SVTPYNQMRYTFGRRVLGDRIVTQGNNNFN 80

As11-Dg24 1 *marf-------nfalacillaavcsa*APEIEKLQ--------ASIEFVDDISAYAAEFPEVDLIPLDSE--VQPFGQIRYTLGGRVAGDRIVAQGNNNFN 83

As11-Dg25 1 *marf-------hitlacillatacaa*APEHDQIG--------LSVEVIDDITEYLAKYPESEITPLRVVVSPIPPFRTRYVLGTRVKGDRVLAIRNDNVF 85

As11-Dg26 1 *myrt---pclvlltlaavfa*FDLDTVQFRAQLLN--------GDIVVVNETTRFQRKYPNVTLNGMMLK-TKLVKGQINYQLGKRIPGDQLVAEKADKQS 88

As11-Dg30 1 *mtalkificaafwavlsiqsadt*SLIFRQIEDLETNNNTLDYSGVETIETHIQLIGENQNEALDQTELT--QNVGVTKYYSLGKRIAGDRVVATGGKAMN 98

As11-Dg1 47 AKTLRVVSEDVQYP--PKGVVGQNLITGIRVTDQYTNGKGGYSTLVAGGPGQRDVTLHFKSQRGHGYNFIVEYYGR* 120

As11-Dg13 46 SKWLRIVTQDYTFP--PPGSVQNRLITGIRVTDQYTNGKGGYASLYAGGPGYNSVTIHLKSQRSQGFKFIVEIFGR* 119

As11-Dg7 46 YKFLRIVTQDYTFP--PPGAVLGRMITGIRVTDQYTNGKGGYASLYAGGPGNPSVTIHFKSQRNHGFNFIVEIFGR* 119

As11-Dg32 44 ASPLRIVTKDVKFG--YKGN--RKVITGIRVIDRMPKQKGGRASLLHGGPGHKNVTIHLKSQRNNGIFFTVEIYGR* 115

As11-Dg36 46 IRHNQVVTKNVLYP--PAGQSQDHTITAIVLTDVHTDGNGGYATLQSGGPGQTHATVHFVSALGHSVNYIMEVYGRRI* 121

As11-Dg15 49 AKKGWEVSENVFYPGIGGGASYDHIFTEIRVTDKCEDGTGGYAYITEGGIGQRSAKIHFISQLSKGYEFLLEIFGH* 124

As11-Dg20 80 YARMQDVRINLNYP--QSGV--GAVITHLDVNLEYDS-NMGKLVLIEGGIGRRTIKIAIEAKAVTHFAANVTLWGV* 150

As11-Dg23 82 YGRMQDVRLNLNYP--QWGV--GAIVTYLEVILEYNS-NMGKLYIVGGGIGQRSIKLVMEANGVTHFAASSTLWGY* 152

As11-Dg22 81 YPQAQDVRTNLNYP--ANGI--GNVVSYVEIFVDQTS-NIGTAYVTSGGIGQRHISIVLEAKRTFRFSYRAAIYGY* 151

As11-Dg24 84 YPTLQDVRLTLNYP--QSGV--GAVVSFVEIVVQQTS-NDGNAYIAAGGIGQRTIRIILEARRTQSFAYRAAIFGY* 154

As11-Dg25 86 YPTRRDIRLDLHYP--QYGT--GGIVTLIDIAVEQSS-DLGRAYIVAGGIGQRQIKFVVEARDVYHLSTNTTIYGY* 156

As11-Dg26 89 FPAPEDIQIVLRYP--TKGE--GATVSHVNIVVDQSS-SVGDGYVIEGGISQKFIGILVEANNTMHFTFSASIYGYY* 160

As11-Dg30 99 WSTPHNVKITLAYP--TRGI--GALVTYVFIAVDQTN-NGGRAYVLNGGVNQRHITVVIEAFGVTHFALAASIYGR* 169
